# Photonic Solutions for Challenges in Sensing

**DOI:** 10.1021/acsomega.4c01953

**Published:** 2024-06-06

**Authors:** Gonzalo Ramírez-García, Lin Wang, Ali K. Yetisen, Eden Morales-Narváez

**Affiliations:** † Biofunctional Nanomaterials Laboratory, Centro de Física Aplicada y Tecnología Avanzada, Universidad Nacional Autónoma de México, 3001, Boulevard Juriquilla, 76230 Querétaro, México; ‡ Department of Chemical Engineering, Imperial College London, SW7 2AZ London, U.K.; § Biophotonic Nanosensors Laboratory, Centro de Física Aplicada y Tecnología Avanzada (CFATA), Universidad Nacional Autónoma de México (UNAM), 3001, Boulevard Juriquilla, 76230 Querétaro, México

## Abstract

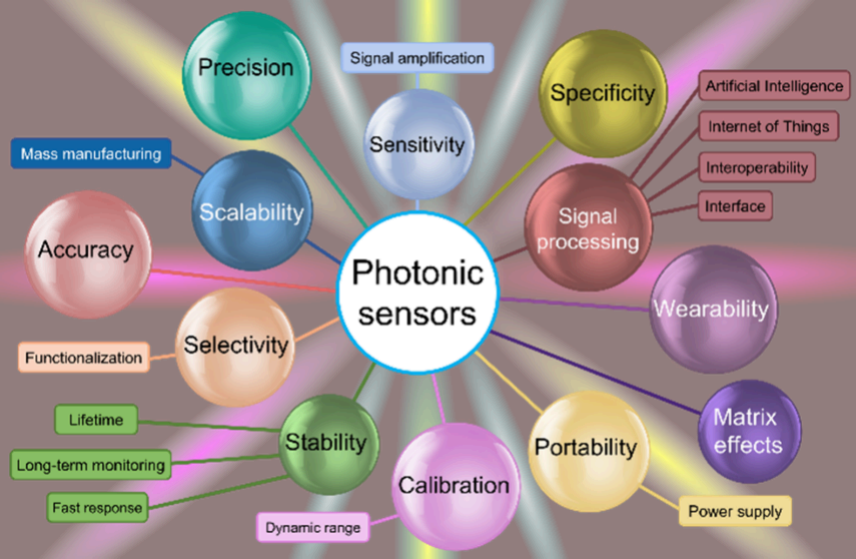

Sensing technologies support timely and critical decisions to save precious resources in healthcare, veterinary care, food safety, and environmental protection. However, the design of sensors demands strict technical characteristics for real-world applications. In this Viewpoint, we discuss the main challenges to tackle in the sensing field and how photonics represents a valuable tool in this sphere.

## Introduction

**Figure 1:**
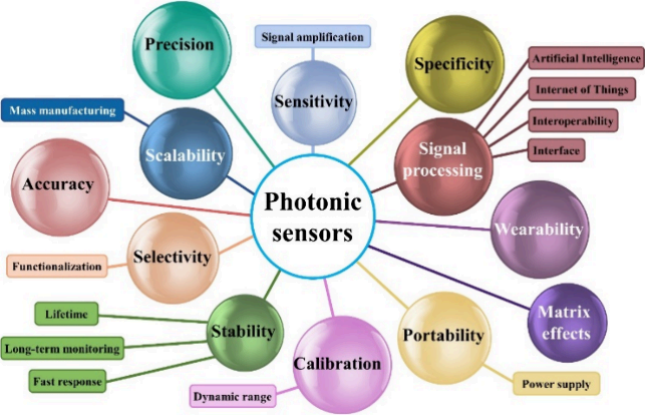
Challenges that should be considered before utilizing sensing devices in real-world applications.

Sensors are analytical devices that can target the control, surveillance, prediction, prevention, and/or management of critical processes. For instance, the lateral flow assay (LFA)-based colorimetric biosensors were significantly utilized to monitor and control the disease spread during COVID-19 pandemic.^1^ Photonic sensors can convert changes in light into electronic signals, providing readable and quantitative information. The utilization of this technology offers one of the most efficient and practical approaches in measurement science.^2^ The synergistic light–matter interactions could support the evolution of signal transducers and interfaces, stimulating the advancement of photonic sensing concepts and unprecedented applications. Photonic sensors are suitable for constructing daily monitoring applications, which can provide rapid, facile, *in situ*, and/or real-time outputs by integrating with conventional laboratory settings or wireless portable and wearable devices.^3^ However, the construction of sensors with high analytical performance still faces challenges due to current technical and inherent sensing principle limitations, which should be addressed prior to being utilized in practical applications and industrial implement. Herein, a brief perspective on both fundamental and essential concepts of photonic sensors is discussed, along with their current limitations and potential solutions (see Figure 1).

## Challenges in the Fundamental Features of Sensors

**Sensitivity** is the ability of a sensing system to detect changes in the target analyte/measurand. Sensors often have a detection limit, which is the lowest detectable level of analytes. The detection limit should be lower enough to improve the sensitivity and thereby supporting sensitive measurements in a required scale. Several real-world applications require the detection of micromolar or nanomolar quantities of the target;^4,5^ for example, pesticides may be present in food, but only at legally permitted levels, such food sensors require the high sensitivity and low detection of limit (LOD). Nanomaterials, such as nanoparticles (NPs), quantum dots (QDs), nanowires (NWs), and nanotubes (NTs) etc., with extraordinary optical and electronic properties at the nanoscale can be further explored to improve the sensitivity of sensors.^6^In addition to utilizing advanced photonic materials, noise reduction (minimizing the noise signal within the sensing system) is critical to achieving high signal-to-noise ratios and thus improved detection limits. For instance, photonic materials emitting light in the biological window (ex. 650 to 950 nm) are known to reduce signal-to-noise ratio and facilitate highly defined biological images.^7^Filtering methods, such as wavelength-induced frequency filtering, also lead to the improvement of signal-to-noise ratio in bioimaging, even allowing for the unprecedented depths of up to 5.5 ± 0.1 cm.^8^

Metal nanoparticles such as gold nanoparticles and silver nanoparticles (AuNPs and AgNPs) have been widely investigated over the past decade for ultrasensitive single-molecule (SM) detection by surface-enhanced Raman spectroscopy (SERS).^9^Metal nanoparticles can concentrate light in nanoscale regions known as hot spots, where SM detection can be carried out via SERS. Detection at the SM level is highly probable in high concentrations of the analyte; however, the lower the concentration of the analyte the less probability to perform SM-SERS. Transdisciplinary efforts (involving experts in photonics, engineering, materials, chemistry, etc.) focused on SM-SERS are required to advance the state of the art of SM-SERS. For example, innovative approaches allowing for remote SERS (SERS carried out not directly within the laser focus) is facilitating new opportunities to perform SM detection even at extremely low concentrations of the analyte, which may bring unprecedented analytical applications; for example, in preventive healthcare (ex. early detection of biomarkers in extremely low concentrations) and environmental protection (ex. detection of traces of pollutants).^10,11^

Recently, a quantitative sensing system that can measure an analyte at clinically or environmentally relevant concentrations has been in high demand. Hence, the **dynamic range** (the range between the smallest measurable signal and the largest measurable signal) offered by a sensing system is expected to operate according to the desired application.^12^

Implementing**signal amplification**elements could enhance the signal strength to allow for small variations detectable, thus increasing the sensitivity. This therefore implies the use of materials that interact with optical label molecules through host–guest interactions. Polymeric or composite materials, offering highly sensitive light-matter interactions, can also be used to amplify signals for the indirect determination of targets. The luminescent nanomaterials (such as QDs), upconverting, and persistent luminescent nanoparticles, etc., have been used as labels to amplify signals in multiple immunochromatographic assays.^13^ However, the generated background signals and potential quenching effects during the amplification process should be considered at the same time.

**Calibration**is essential in developing photonic sensors as it can ensure the accuracy and precision by the establishment of a known relationship between the sensor outputs and measurements being conducted. The instability, susceptibility, and complexity of samples in a multivariate environment could lead to false negative or positive readings. Fluctuations in ambient condition parameters and exogenous compounds can results in fouling effects, chemical alteration, and nonspecific adsorption at the interface of sensors, affecting calibration and the overall analytical response.^14^**Accuracy** quantifies the sensor’s ability to consistently provide reliable data, ensuring it yields values that are accepted or expected, whereas **precision** evaluates the dispersion of data produced by sensors. In photonic sensors, these parameters could be improved by utilizing fluorophore–quencher pairs, which can modulate the optical response and the concentration-signal correlation. Moreover, ratiometric strategies allow self-calibration by monitoring two or more absorption or emission bands to compensate for variations or effects derived from the environment. For example, thermally activated delayed fluorescence (TADF) and fluorescence (FL) were simultaneously induced in organic molecules devoted to the detection of the local polarity variation in phospholipid systems mimicking membranes. While the TADF acts as a sensing signal with both wavelength and lifetime that correlate with polarity, the FL remains constant for internal referencing.^15^ It is worth discussing that LOD, dynamic range, accuracy, and precision are often evaluated by experts in sensing to determine and compare sensors performance.

**Specificity** is the capability of sensors to offer reliable analytical signals in the presence of a single target, avoiding interference from other possible stimuli and environmental conditions. Hence, nonspecific interactions between the sensor surface and molecules other than the targets could be avoided by modifying surface (ex. with blocking agents) or optimizing receptors, thus increasing the sensor’s specificity.**Selectivity** is displayed by those sensing systems designed to determine several targets independently from each other.^16^ Real-world scenarios require the detection of biomolecules and chemical species with high specificity and selectivity, thereby avoiding false positive results. In biosensing or chemosensing systems, specificity and selectivity could be achieved through recognition elements or ligands that exclusively bind to the target molecule.^17−19^ The major limitation is the difficulty in obtaining high-performance biorecognition elements against small-molecule targets, where novel extraction and synthetic methods should be explored.^20^ Recently, aptamers (single stranded DNA or RNA molecules) are emerging biorecognition elements that can be used in the pursuit of high affinity and specificity with several advantages (e.g., ease of synthesis, rapid and inexpensive production, less batch-to-batch variations, and stable for transportation etc.) compared to conventional antibodies.^21,22^**(Bio)functionalization** or the development of a specific surface chemistry operating as a recognition element is also challenging due to unspecific interactions.^23−25^ However, the development of optical sensors, where the detection process occurs in the liquid phase, can avoid surface functionalization, among other cumbersome procedures employed in biosensing, including washing, separation, or blocking procedures.^26^ Although this approach is feasible for *in vitro* diagnostics, it is not particularly suitable for *in vivo* testing, which generally requires real-time and continuously monitoring.

**Stability** is the sensor’s ability to deliver consistent results under the same circumstances over time. Various factors, such as the sensor inherent design, environmental interferences, and handling process, could lead to its decreased stability, while it is essential to maintain a good function of the sensor in a long-term period to ensure its reliable and consistent performance. The overall performance of sensors can be affected by biofouling, chemical changes, irreversible adsorption, material degradation, and mechanical failure. The continuous exposure to biofluids or complex matrixes usually triggers such undesired effects; even worse, sensors are susceptible to degradation even when in idle mode. The challenges for **sensor lifetime** include the degradation of sensor performance over time due to aging effect, contamination, corrosion of materials, and changes in properties caused by chemical or physical variables such as temperature and humidity. The optimization of the fabrication and utilization of biomimetic materials (functionalizing a sensing surface) that are resistant to denaturation and degradation, such as nanozymes, synzymes, infinite coordination polymers, nanochannels, metal complexes, and molecularly imprinted polymers (MIPs) are suitable alternatives to overcome the aforementioned limitations^20,27^ Another strategy involves the implementation of surface modifications or coatings on the sensor materials to protect them from degradation and adsorption of undesired species. For instance, an inner salt, zwitterion, has been implemented as a brush layer to prevent fouling by proteins, making nanophotonic substrates suitable for clinical applications such as anticancer drug monitoring in serum.^28^

Potential interferences or modifications of the analytical performance caused by **matrix effects** should be carefully considered during the development of sensors.^20^ Those include physical and mechanical factors that can introduce spectroscopic noise or undesirable background signals. In practical terms, the high costs associated with extraction and purification procedures limit the commercial viability of a sensor. To overcome these constraints, luminescent probes have been used to enhance the signal-to-noise ratio through *turn-on* or *turn-off* schemes.^29^ Sensors incorporating colorimetric and fluorescent dual-mode sensing probes have also been developed, as well as sandwich assays, where the analyte is bound to a recognition molecule and a complementary labeled recognition molecule is bound to the analyte, thus avoiding potential interferences.^30^ Furthermore, lifetime measurements provide a robust alternative, as they are less prone to interferences, utilizing pulsed excitation with single photon counting or phase modulation. However, the instrumentation required for measuring average lifetimes is still complex and not amenable to portable applications. Nonetheless, the use of ratiometric methods such as dual-wavelength rationing or dual lifetime referencing is under development to improve intensity-based measurements of luminescent sensors.

## Challenges in the Next Generation of Sensors

**Monitoring of biological processes** in living subjects is pivotal to advance the state-of-the-art biomedical research areas such as physiology, pharmacology, toxicology, and personalized medicine. In this challenging bioanalytical field, there is often a need for reversible, continual, real-time and/or long-lasting measurements.^31^ Furthermore, sensors face the challenge of finding suitable body regions for their applications as they need to overcome the inherent difficulties presented by natural barriers. The skin may operate more as an information barrier rather than an information source, which presents significant challenges for wearable sensors.^32−34^In this context, sweat as an emerging noninvasive body fluid attracts more research interest, while the main limitation of developing sweat-based wearable sensor could be the unreliable readings due to its variability and complexity. Reversible near-infrared fluorescence probes are being developed to monitor biological processes in real-time, even in deep tissues.^35^ Optically active functional fragments of molecules (e.g., azobenzene moiety) connected with biorecognition elements such as aptamers can provide biosensing surfaces with regeneration capabilities; particularly, when specific wavelengths (e.g., UV light) illuminate the sensing surface, thereby offering reversibility in biosensors.^36^ Meanwhile, optical fibers represent an attractive platform for *in vivo* biosensing.^37,38^ For instance, optical fibers integrated with microneedles may lead to innovative wearable sensors that could support real-time and/or long-term monitoring. However, biocompatibility should also be considered for body-worn or implantable applications.

Timely decisions aimed at taking corrective/preventive actions may depend on the **fast response** of a sensing system.^33,34^ Photonic sensing mechanisms can offer a quick analytical response. For instance, photonic sensors can detect gases in less than one second, (bio)molecules within 15 min^39^ or physical parameters such as temperature in a few seconds.^40^ The challenges in **measurement duration** (hours instead of days) or **long-term monitoring** limit the real-time analytical ability of sensors and the need to obtain single measurements through an on-demand or continual manner. Furthermore, current analytical techniques such as optical and fluorescence microscopy, which only allow for single measurements, involving the utilization of labels that can interact nonspecifically with cells and substances under test. Performance issues such as signal intensity, stability, and interference with cultured cells should also be addressed.^34^ Therefore, ensuring the stability of the sensing elements over extended periods is crucial for long-term measurements. For instance, the integration of flexible optical elements into a wristband allows for the protection of components in a wearable interrogator, while the fibers might be broken under some extreme conditions. Hence, flexible polymer optical fibers are more amenable to integration with soft textiles. The instability of light source chips is also a challenge that could be solved by implementing C-band semiconductor lasers on functional substrates.^41^

Sensors are currently sought to be **portable**, easy to use and interpret, even for nonexperts for point-of-care testing applications.^42^ Photonic sensors have been equipped with automated operation and built-in sensorgram analysis software and tools allowing for easy miniaturization and portability. The integration of different building blocks involving flexible/stretchable photonics,^43,44^ microfluidics, small circuit chips, liquid-crystal displays, smartphone-based interfaces, artificial intelligence, and connectivity with the Internet of Things will facilitate the user-friendliness of sensors. Advances in smartphone technology have rendered them suitable as readout devices for the field use of biosensors, providing portability and processing power. This enables quick feedback into process control and the rapid interpretation of multiplex sensors.

An increasing energy demand exists in our contemporary world; therefore, achieving low power consumption is pivotal yet challenging in next-generation sensing technologies.^45^ The integration of sensors with **power supply** components allows for wireless monitoring and detection. Silicon photonics as well as 2D materials-based optics are at the frontiers of low-power optoelectronic detectors,^45,46^ mainly to perform communication tags, which could be integrated/applied in sensing systems. Self-powered sensors based on the piezoelectric effect, triboelectric effect, or moisture-driven generators are also emerging to relieve the dependence of sensors on an external power supply.^47^

**Wearable sensors** have been integrated with existing mechanical, electrical, and optical anthropometric methods, leading to commercial progress in the field. This integration involves innovations in miniaturizing sensing technologies, thus providing conformal and flexible, and developing companion software to enhance the value of measurement data. Optical sensors are designed to capture information by introducing light into the body through the skin. The body reveals information through changes in light scattering and absorption, which are then interrogated by optical detectors. Light sources utilized in these sensors range from broadband incoherent lamps to narrow-band LEDs and lasers. Detectors can include broadband photodiodes, avalanche photodetectors, and photomultiplier tubes. Various passive devices such as integrated optics, diffraction gratings, optical filters, and lenses are also used for light capture, wavelength selection, and light guidance.^32^ The limitations in the **sensor interface** and **signal processing** also pose challenges for portable and wearable sensor implementation. For instance, the signal processing algorithm should be robust enough to extract useful information from noisy interferences, indicating it requires to be sensitive to the variation of environment and individual differences. Several unresolved challenges and outlooks for photonic-based sensors require the development of low-power computational capabilities for data analysis. In addition, specificity/selectivity can be achieved through the **artificial intelligence** analysis of optical/spectral signals.^48−50^ Enhanced communication capabilities play a vital role in establishing multinode sensing networks that can acquire spatial information from different points across the sample. This could provide a more comprehensive understanding of the concentration and distribution of the analyte being measured.

Recently, the **Internet of Things (IoT)** and telemedicine connectivity in wearable sensors are driving the field of remote health monitoring to evolve point-of-care platforms.^51^ These sensors can be integrated with wireless connectivity, which enables the acquisition of real-time physiological and biochemical data for early disease diagnosis and daily monitoring of patients. Wearable devices may require more processing memory than the current microprocessor can provide to sustain artificial intelligence operation for big data execution. Strategies to reduce the amount of operation memory stored in the microprocessor while improving artificial intelligence algorithms or integrating smart materials with sensing multifunctionality are being explored.^23,52^

**Interoperability** of sensors can be implemented through connectivity with the IoT, where secure data storage and transfer remains a concern. The immutability, decentralized and smart character of blockchain, as well as the technology behind cryptocurrency, could facilitate robust security systems to ensure the confidentiality and integrity of patient data.^53^

The optical and photonics sensing modalities have also experienced challenges in **large-scale applications**, especially for noninvasive or minimal-invasive sensors. The industrial mass manufacturing of sensors represents an unsolved challenge for emerging sensing technologies. The transition from laboratory-fabricated sensors to industrial-scale manufacturing requires more effort on all fronts. For example, ensuring consistent sensor performance and quality in large-scale production requires the development of scalable manufacturing processes that maintain tight control of critical parameters. Challenges and opportunities for the commercialization of sensors are the complexity and natural variation in relevant samples. This makes it difficult to develop sensors that can be reliably utilized across all samples of the same type. Furthermore, commercial sensors should consider local governing legislations and regulatory landscape; for example, safety, sensitivity, dynamic range, and calibration should meet permissible parameters established by those regulations.

Challenges for **mass manufacturing** of sensors include the high cost of development, limited market demand, and the requirement for longer development cycles to fulfill performance requirements and validation procedures. Photonics has been making noteworthy progress in terms of both performance and capability. Currently, numerous manufacturing facilities and foundries are equipped with cutting-edge passive and active components such as modulators, photodetectors, and lasers.^54^However, next generation of sensors are also expected to consider sustainability (greenness of the approach) in their design and manufacturing process.^55^

## Conclusion

Photonic sensors have a wide range of applications in healthcare, environmental monitoring, food quality assessment, veterinary testing, and biochemical process assessment. Therefore, a deeper understanding of fundamental challenges faced in highly sensitive, selective, portable, or wearable devices is necessary to drive the creation of the next generation of sensor innovations and breakthroughs. In real-world applications, sensors must exhibit high sensitivity to detect low concentrations of analytes within complex matrices while also being capable of differentiating between the target analyte and interferents present in the sample, ensuring probe stability and measurement reproducibility. Typically, samples need to be prepared and concentrated before analysis, and sensors must be user-friendly in field applications. Thanks to the progress in electronics, photonic sensors can now be incorporated into portable devices such as lab-on-chip, lateral-flow assays, textiles, food packaging, and different wearable arrays and even implemented in living organisms. Such photonic devices are expected to be suitable for deployment in remote areas and resource-constrained environments. Furthermore, those systems have been integrated with other platforms such as the Internet of Things, big data, and artificial intelligence to enable large-scale networking and facilitate the interpretation of the results. The intersection of advances in photonics and electronics signifies that the copackaging of these two technologies allows for the ongoing development of both domains, driving further innovation in the field of sensors. Collaborations between academic and research institutions, medical facilities, and industrial device prototyping facilities are crucial to advance the quality and industry standards of sensors, thus ensuring their readiness for commercial deployment. All in all, the next generation of photonic sensors will facilitate a new era of environmental protection and healthcare (personalized, noninvasive, preventive, etc.).
